# Ultrafast emergence of ferromagnetism in antiferromagnetic FeRh in high magnetic fields

**DOI:** 10.1038/s44306-024-00069-6

**Published:** 2025-02-03

**Authors:** I. A. Dolgikh, T. G. H. Blank, A. G. Buzdakov, G. Li, K. H. Prabhakara, S. K. K. Patel, R. Medapalli, E. E. Fullerton, O. V. Koplak, J. H. Mentink, K. A. Zvezdin, A. K. Zvezdin, P. C. M. Christianen, A. V. Kimel

**Affiliations:** 1 Radboud University, Institute for Molecules and Materials, 6525 AJ Nijmegen, The Netherlands; 2 High Field Magnet Laboratory (HFML - EMFL), Radboud University, Toernooiveld 7, 6525 ED Nijmegen, The Netherlands; 3 Interactive Fully Electrical Vehicles Srl, 100040 La Loggia TO, Italy; 4 Center for Memory and Recording Research, University of California San Diego, La Jolla CA, 92093-0401 USA; 5 Institute of Problems of Chemical Physics, Chernogolovka, Russia; 6 Istituto P.M. Srl, 10138 Turin, Italy; 7 The Lebedev Physical Institute of the Russian Academy of Sciences, 119991 Moscow, Russia; 8 Present Address: University of Milan-Bicocca, 20126 Milan, Italy

**Keywords:** Ferromagnetism, Magnetic properties and materials, Phase transitions and critical phenomena, Spintronics

## Abstract

Ultrafast heating of FeRh by a femtosecond laser pulse launches a magneto-structural phase transition from an antiferromagnetic to a ferromagnetic state. Aiming to reveal the ultrafast kinetics of this transition, we studied magnetization dynamics with the help of the magneto-optical Kerr effect in a broad range of temperatures (from 4 K to 400 K) and magnetic fields (up to 25 T). Three different types of ultrafast magnetization dynamics were observed and, using a numerically calculated H-T phase diagram, the differences were explained by different initial states of FeRh corresponding to a (i) collinear antiferromagnetic, (ii) canted antiferromagnetic and (iii) ferromagnetic alignment of spins. We argue that ultrafast heating of FeRh in the canted antiferromagnetic phase launches practically the fastest possible emergence of ferromagnetism in this material. The magnetization emerges on a time scale of 2 ps, which corresponds to the earlier reported time scale of the structural changes during the phase transition.

## Introduction

**Fig. 1:**
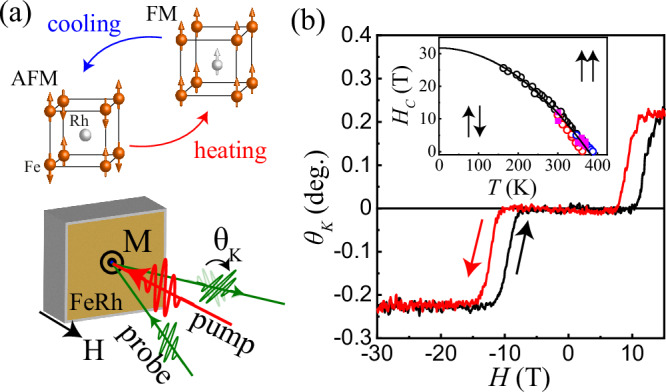
Magneto-structural phase transition in FeRh between AFM and FM phases. **a** (top) Schematic representation showing the magneto-structural phase transition in a FeRh unit cell. FeRh is a metallic compound with a CsCl crystal structure. At low temperatures, in the absence of any external fields and at ambient pressure, the spins of the Fe atoms are aligned antiferromagnetically and have a net magnetic moment of m_Fe_≈ 3μΒ per atom, while the Rh atoms have no magnetic moment^57^. Above 370 K, The parallel aligned magnetic moments of iron remain at m_Fe_≈ 3 μ_B_ per atom, while the Rh ions acquire an atomic magnetic moment of ${{\rm{m}}}_{{\rm{Rh}}}\approx {1{\rm{\mu }}}_{{\rm{B}}}$ ^58,59^. (bottom) Illustration showing the time-resolved p-MOKE geometry used in the experiments. **b** p-MOKE loop measured at 300 K in the absence of a pump. Arrows indicate the direction of the magnetic field sweep. (inset) Critical magnetic field, ${{\rm{H}}}_{{\rm{C}}}$ that triggers the phase transition in Fe_50_Rh_50_. The black solid line represents a fit to all literature data to the expression ${{\rm{H}}}_{{\rm{C}}}({\rm{T}})={{\rm{H}}}_{{\rm{C}}}(0)\left(1-{\left(\frac{{\rm{T}}}{{{\rm{T}}}_{{\rm{c}}}}\right)}^{2}\right)$ as proposed in ref. ^9^ using ${{\rm{T}}}_{{\rm{c}}}=378\pm 2$ K and ${{\rm{H}}}_{{\rm{c}}}(0)=31.7\pm 0.6$ T. The open circles illustrate previously obtained results of the critical magnetic field of the transition for samples of similar composition: black^9^, red^30^, and blue^31^. The magenta squares represent the SQUID (see Supplementary Materials Fig. S1) and p-MOKE data for our sample as explained in the text. Arrows indicate the spin phase in FeRh.

The metallic alloy of iron and rhodium (FeRh) stands out for its counter-intuitive heat-induced ferromagnetism, first reported in 1938^1^. Equiatomic FeRh exhibits an antiferromagnetic (AFM) phase at lower temperatures and becomes ferromagnetic (FM) when heated above 370 K (see Fig. 1a). This magnetic change is accompanied by an expansion of ~1% in the FeRh unit cell. The nature of this heat-induced ferromagnetism in FeRh has been a subject of debates for nearly 60 years. The central question - whether the change in the magnetic order drives the lattice expansion or vice versa - resembles the classic chicken-or-egg causality dilemma^2–6^.

The earliest hypothesis suggested that this is a structural phase transition, where the structural change leads to a sign change of the exchange integral, thereby initiating a transition in the Fe-spin order from AFM to FM^7^. However, later it was argued that this mechanism is inconsistent with the actual change of the lattice entropy. The latter, estimated from experimental data, appeared to be much less than the total entropy change^8^. Several computational studies have proposed that the magneto-structural phase transition is entirely driven by processes within the spin system^9–13^. At the same time, recent calculations suggest that the changes in lattice entropy and the total entropy differ only by a factor of 3^14^ or are even comparable^3^. Attempts to detect the ultrafast kinetics during the phase transition using a femtosecond laser pulse – serving as ultrafast heater- and monitoring the laser-induced dynamics of the lattice and spins yielded conflicting results. While some studies^15–19^ claim that the changes in the spin system occur more rapidly than those in the lattice, others^20–22^ claimed that the lattice expansion precedes the emergence of net magnetization. It was even found that the electronic band structure changes on a sub-ps time-scale during the laser-induced transition, far before either the lattice or spins respond^23^. In addition to these fundamental challenges, in practice, the study of FeRh is hampered by the coexistence of the low-temperature AFM and the high-temperature FM phases^24–26^. Although such coexistence is very typical for first-order phase transitions, it often complicates the interpretation of experimental data, as discussed in ref. ^27^. Thus, the sequence and the underlying mechanism of the magnetic and structural dynamics in the magneto-structural phase transition of FeRh remain unclear and further progress in the field crucially depends on our ability to obtain novel insights into the phenomenon with the help of new experimental techniques.

Here we propose to employ high magnetic fields as a new variable in experimental studies of the kinetics of the phase transition. In particular, we investigate the laser-induced phase transition using the time-resolved magneto-optical pump-probe technique across an unprecedently broad phase space of temperature (*T*) (from 4 K to 400 K) and magnetic field strength (*H*) (from 0.125 T to 25 T). Our experiments confirm that, depending on *T* and *H*, the femtosecond laser pulse can trigger distinctly different ultrafast magnetization dynamics in FeRh. Based on the calculated *H-T* phase diagrams, we attribute this variation to three different initial spin order states in FeRh: collinear AFM, canted AFM, and FM phases. Contrary to the commonly accepted wisdom that spin dynamics, in general, and magnetic phase transition, in particular, are accelerated in an applied magnetic field^27–29^, our experiments in fields up to 25 T reveal that in FeRh the fastest characteristic time in the kinetics of the transition from the canted AFM to the FM phase is field independent and can never get faster than 2 ps. Hence, our measurements set a benchmark for the ultimately fast emergence of ferromagnetism in antiferromagnetic FeRh.

## Results

The sample studied here was a 40 nm thick FeRh grown on MgO single crystal substrate and capped with a 5 nm thick Pt layer as described in the Methods section. It is known that an external magnetic field shifts the critical temperature at which the magneto-structural phase transition in FeRh occurs^9^. Figure 1b shows the hysteresis loop measured from the studied FeRh using the static polar magneto-optical Kerr effect (p-MOKE) at 300 K and subtracting the signal that scales linearly with the applied magnetic field. The origin of the observed hysteresis is the phase transition from the AFM to the FM phase. The critical magnetic fields, *H*_*C*_ at which the transition occurs can be estimated from discontinuities of the first derivative of the observed field dependence. The fields deduced from our measurements fit well to the temperature dependence of *H*_*C*_ published earlier for FeRh of the same or closely similar composition^9,30–32^ and the summary of the data is shown on the inset of Fig. 1b. This consistency shows that our p-MOKE measurements reliably probe the phase transition from the AFM to the FM state in FeRh.

**Fig. 2:**
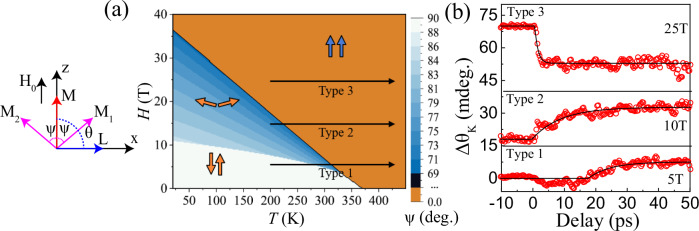
Three different types of ultrafast magnetization dynamics in FeRh. **a** (left) Coordinate system used for the numerical calculations of the free energy. Here, ψ is the angle between ${{\rm{M}}}_{{\rm{i}}}$ and the magnetization ${\rm{M}}=({{\rm{M}}}_{1}+{{\rm{M}}}_{2})/2$; θ is the angle of the antiferromagnetic vector ${\rm{L}}=({{\rm{M}}}_{1}-{{\rm{M}}}_{2})/2$ with respect to the z-axis. ${{\rm{H}}}_{0}$ denotes the direction of the external magnetic field. (right) Numerically calculated *H-T* phase diagram using the thermodynamic potential described in the text: light blue region – collinear AFM phase, dark blue region – canted AFM phase, brown region – FM phase. The horizontal black arrows schematically illustrate the three different types of ultrafast pathways into the FM phase at 200 K, depending on whether the initial phase is collinear AFM (type 1), canted AFM (type 2) or FM (type 3). **b** The laser-induced change in the probe polarization rotation, Δθ_K_ (red circles) for *H* = 5 T, 10 T, and 25 T corresponding to routes of types 1, 2, and 3, respectively, measured at *T* = 200 K. The solid black lines are the fits to the data. For the route of type 1, a latency appears in the dynamics, similar to ref. ^27^ which is estimated to be $\Delta {\rm{\tau }}=17.0\pm \,0.6$ ps.

Even though FeRh *H-T* phase diagrams, distinguishing the regions separating two different spin phases – collinear AFM and FM such as the one shown in the inset of Fig. 1b were discussed several times before^9,12,30–39^, it is clear that this diagram is incomplete. If a magnetic field *H* is applied perpendicular to the antiferromagnetic spins, the field cants spins over an angle defined by the ratio of the applied magnetic field to the effective field of the exchange interaction between the spins^40^. The canting angle increases with increasing *H*. If the magnetic field is applied parallel to the antiferromagnetically coupled spins, these spins remain insensitive to the field until the so-called spin flop field ${H}_{{\rm{sf}}}$ is reached. Very recently, Buzdakov et al.^41^, numerically calculated *H-T* phase diagrams in vicinity of the magneto-structural phase transition in FeRh. This theoretical framework supported by XMCD measurements allowed to identify a number of possible phase diagrams. It was shown that next to the collinear AFM and FM phases a canted AFM phases must exist. Using the same approach, we calculated numerically the *H-T* phase diagram for the geometry as shown in Fig. 2a (see Methods for a full description of the numerical model). We note that the order of a phase transition between the AFM (either canted or collinear) and FM phase can change upon varying *H* and *T*. However, it has been shown that the most probable phase diagrams for FeRh implies that the phase transition is of the first order at every magnetic field *H* and temperature *T*^41^. The phase diagram hosts three different phases – FM, collinear AFM and canted AFM phase. The horizontal black arrows in the right panel of Fig. 2a show that by properly choosing the values of *H* and *T*, one should be able to stabilize different phases and launch different heat-induced transitions between different phases. For instance, at *T* = 200 K and *H* = 5 T, heating over a range of 230 K should cause a phase transition from the collinear AFM to the FM phase (denoted by the route type 1 arrow). At *T* = 200 K and *H* = 10 T, the same heating should trigger a transition from the canted AFM to the FM phase (route type 2). Finally, at 200 K and the highest field *H* = 25 T, the heating would partially destroy the FM order leading to ultrafast demagnetization (route type 3).

Figure 2b shows ultrafast dynamics of the p-MOKE triggered in FeRh by intense femtosecond pump pulses, as described in Methods section. The transients detected at *T* = 200 K in magnetic fields of 5, 10, and 25 T, respectively, reveal three different types of laser-induced spin dynamics. At *H* = 5 T, the p-MOKE signal exhibits a profound latency *Δτ* similar to ref. ^27^ before starts to rise (similar behaviour can be observed at higher temperatures, see Supplementary Materials Figs. S2 and S3). The polarization rotation can be fitted using the function,

1 $$\varDelta \theta (t)={B}_{M}\left({1-e}^{-\frac{t-\varDelta \tau }{{\tau }_{M}}}\right)$$

Here, *B*_M_, τ_M_, and *Δ*τ are the amplitude, characteristic time corresponding to the laser-induced magnetic changes, and latency time, respectively. The fits to the corresponding data are shown in Fig. 2b. From the fit of the data obtained at *H* = 5 T, we find that ${B}_{{\rm{M}}}\, >\, 0$ and $\varDelta \tau =17.0\pm 0.6$ ps. At *H* = 10 T, the observed dynamics is substantially different and no latency is seen (${B}_{{\rm{M}}}\,>\, 0$, *Δ*τ = 0). The magnetization starts to grow immediately after the pump excitation. Further increase of *H* up to 25 T substantially changes the dynamics. The amplitude changes sign$\left({B}_{M}\,< \,0\right.$) and the dynamics acquire characteristic features of ultrafast demagnetization ($\varDelta \tau =0$). It is natural to attribute these three types of dynamics to the three types of routes as indicated in Fig. 2a. In this case, the type of the observed dynamics must be fully defined by the *H-T* phase diagrams.

**Fig. 3:**
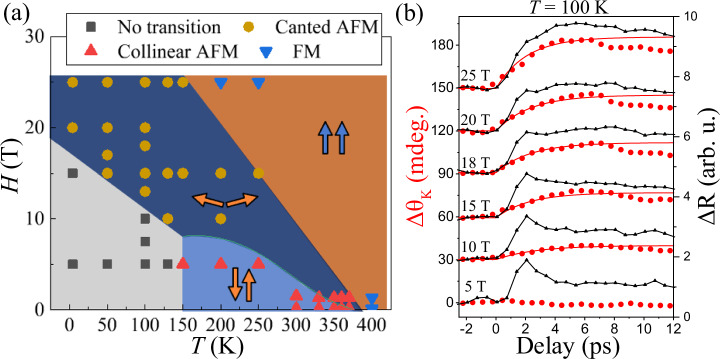
Phase diagram deduced from laser-induced p-MOKE dynamics. **a** Every time-resolved trace was assigned to a certain route type of ultrafast dynamics depending on the sign of the amplitude ${B}_{{\rm{M}}}$ and the presence of latency ($\Delta {{\tau }}$). In the blue area (and Δτ > 0) laser excitation triggers dynamics along Route 1 (red triangles). In the dark blue area (${B}_{{\rm{M}}}\,>\,0$ and Δτ = 0), the observed dynamics is assigned to Route 2 (brown circles). In the brown area (${B}_{{\rm{M}}}\,<\,0$ and Δτ = 0), the dynamics are assigned to Route 3 (blue triangles). In the grey area, the dynamics showed too low amplitudes, which could not be reliably assigned to any of these three types (black squares). **b** The laser-induced dynamics of Δθ_K_ (solid red circles) for various *H* values and *T* = 100 K are shown. The simultaneously measured normalized ΔR dynamics (solid black triangles) are also shown. Solid curves (red) are the fits to the experimental Δθ_K_ data. The curves corresponding to Δθ_K_ for different (*H*, *T*) cases and the ΔR dynamics are plotted with an offset along the y-axis for clarity.

To verify this hypothesis, we measured the laser-induced dynamics for various combinations of (*H*, *T*) values. Each of the observed transients has been assigned to one of the three types: type 1 (${B}_{{\rm{M}}}\,>\,0$, *Δτ* ≠ 0), type 2 (${B}_{{\rm{M}}}\,>\,0$, *Δτ* = 0) and type 3 (${B}_{{\rm{M}}}\,<\,0$, *Δτ* = 0). To simplify the assignment procedure, we neglect changes of Δθ_K_ less than 10% of the maximum demagnetization signal. For instance, the dynamics obtained at 200 K and 5 T, as shown in Fig. 2b, have been attributed to type 1, even though between 0 ps and 18 ps the signal is strictly speaking not zero. The results of the classification are shown in Fig. 3a, where one can clearly distinguish three different regions for those cases when the dynamics exhibited a sufficiently high amplitude. The resulting regions with routes of type 1, 2, and 3 are in close agreement with the regimes where we expected to find collinear AFM, canted AFM, and FM starting phases (see Fig. 3a), respectively.

In order to reveal the fastest possible AFM-FM phase transition, one should focus on the dynamics without latency by exploring the type 2 route in ever-higher magnetic fields. Figure 3b presents a selection of typical time traces of the p-MOKE signal (solid red symbols) measured at 100 K and in magnetic fields up to 25 T. While the p-MOKE transients measured at 10 T or below, are characterized either by zero or insignificant (<10%) response, the dynamics changes dramatically at higher fields (*H* ≥ 15 T). Fitting the p-MOKE dynamics with the same exponential function (Eq.1) reveals that the time required to reach a quasi-equilibrium, and thus the characteristic time ${\tau }_{{\rm{M}}}$, is largely field independent for *H* ≥ 15 T. The average rise time for fields above 15 T gives ${\tau }_{{\rm{M}}}=2.2\pm 0.4$ ps. Using the fits shown in Fig. 3b, we have also extracted the *B*_*M*_ values corresponding to the amount of the net magnetization induced in the sample (see Supplementary Materials Fig. S4). The amplitude can also be extracted simply from the raw data taking the signal at the moment when the dynamics have reached a quasi-equilibrium at 6 ps. Figure 3a, b, clearly demonstrates that varying *H* can substantially accelerate the dynamics triggered by ultrafast heating. Note that at lower fields and temperatures, transients could not be assigned to any of the three routes (black squares in Fig .3a). We believe that, in this case, the pump pulse does not provide enough heat to reach the FM phase.

## Discussion

Interestingly, if the heating is ultrafast and realized with the help of fs-laser pulses^15,16^, each of the aforementioned three routes must have distinctly different kinetics. Indeed, the dynamics of the magnetic moment **S** associated with spin in the effective exchange field of its neighbouring spins ${{\bf{H}}}_{{\rm{E}}}$ must obey the fundamental law of conservation of angular momentum $\frac{d{\bf{S}}}{{dt}}=-\gamma [{\bf{S}}\times {{\bf{H}}}_{{\rm{E}}}]$, where *γ* is the gyromagnetic ratio. When the system is in the collinear AFM state, an instantaneous change of the sign of the effective exchange interaction ${{\bf{H}}}_{E}$ does not immediately launch spin dynamics. Since the spins are collinear, the torque acting on the spin is zero $\left[{\bf{S}}\times {{\bf{H}}}_{{\rm{E}}}\right]=0$, and starting the spin dynamics requires an additional trigger. This additional requirement results in a latency *Δτ* as shown in ref. ^27^. When the antiferromagnet is in a canted state $[{\bf{S}}\times {{\bf{H}}}_{{\rm{E}}}]\,\ne \,0$ and a change of the exchange interaction will instantaneously launch the spin dynamics. Therefore, it is clear that the kinetics of the phase transition to the FM phase from the collinear and from the canted AFM phases must be substantially different. This distinct kinetics further reinforces the likelihood of a canted AFM phase in FeRh, as suggested by numerical calculations^41^. One might argue that such a canting phase should manifest visible in static p-MOKE signals. However, this would only be the case if the canting angles were sufficiently large (>1 deg.) to provide a p-MOKE signal above the experimental noise level. Finally, if FeRh is already in a FM phase, ultrafast heating with the help of a femtosecond laser pulse results in ultrafast demagnetization^42^. While modelling ultrafast magnetization dynamics in FeRh remains to be a challenge, it is possible to show that a sudden change of the exchange interaction in collinear and canted AFM state triggers substantially different dynamics even in a simple case of two antiferromagnetically coupled macrospins. Such simulations are described in Supplementary Information (see Supplementary Materials Fig. S6 and the corresponding text). It is shown that applying the magnetic field above the spin-flop value qualitatively changes the spin dynamics. Below the spin-flop field, after an ultrafast change of the exchange field ${{\bf{H}}}_{{\rm{E}}}$ the spin dynamics takes off slowly and get accelerated at longer time delays. Above the spin flop field, a sudden change of ${{\bf{H}}}_{{\rm{E}}}$ launches the spin dynamics immediately. Showing that the dramatic changes in the magnetization dynamics upon crossing the spin-flop field are intrinsic even to the simplistic two-spin model, suggests that this behaviour is a general feature of all AFMs, not just FeRh.

In the very first time-resolved studies of the laser-induced ferromagnetism in antiferromagnetic FeRh, it was suggested that ultrafast lattice relaxation resulting from a magneto-structural phase transition in FeRh can be monitored by measuring ultrafast reflectivity changes ΔR (ref. ^15^). Typically, a change in reflectivity is observed due to a change in the sample temperature, strain in the sample via the laser-induced acoustic phonon generation and most importantly from the lattice expansion. As explained in ref. ^15^ the obtained ΔR transients contain contributions from the electronic part and the lattice part. For instance, the rapid increase and relaxation of the reflectivity signal within the first 2 ps after ultrafast laser excitation are conventionally assigned to be due to ultrafast heating and cooling of free electrons. The slower contribution, that takes place on ps to tens of ps can be referred to the processes of lattice dynamics. It is thus interesting to compare the p-MOKE transients with those of the transient reflectivity.

Figure 3b also shows the transient reflectivity in comparison with the p-MOKE transients discussed above. It is remarkable that if the dynamics is triggered at the field and the temperature corresponding to the canted AFM phase, both the p-MOKE and reflectivity reach quasi-equilibrium values practically simultaneously. We fitted the transient reflectivity with a similar expression as for the magnetization dynamics (Eq. 1),

2 $$\frac{\Delta R}{R}\left(t\right)\,={A}_{{\rm{L}}}\left(1-{e}^{-\frac{t}{{\tau }_{{\rm{L}}}}}\right)$$

where A_L_ and *τ*_L_ are the amplitude and the characteristic time presumably corresponding to the laser-induced lattice expansion. Also for the reflectivity data, it is seen even from the raw data that the rise time *τ*_L_ does not depend on *H* (see Supplementary Materials Fig. S5). Again, the amplitudes can also be extracted simply from the raw data taking the signal at the moment when the dynamics has reached a quasi-equilibrium at 6 ps.

**Fig. 4:**
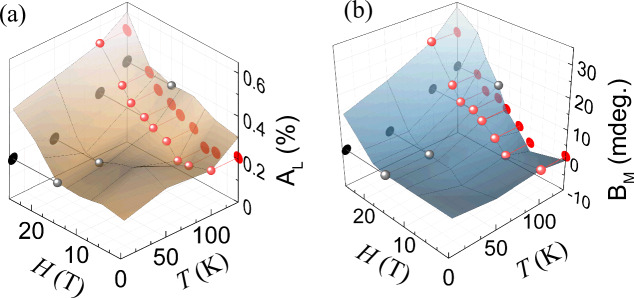
Three-dimensional (3D) surface plots showing the amplitudes A_L_ and B_M_ of ultrafast magneto-structural changes. Plane curves correspond to a slice of the surface taken at the fixed temperature *T* = 100 K (red) or the fixed magnetic field *H* = 15 T (gray). **a** The amplitude of p-MOKE dynamics *B*_M_ reveals ultrafast laser-induced magnetization. **b** The amplitude of ultrafast laser-induced reflectivity change *A*_L_ is presumably assigned to the lattice expansion.

If the observed ΔR and Δθ_K_ dynamics correspond to the magneto-structural phase transition, the amplitudes *A*_L_ and *B*_M_ must be proportional to the probability of the nucleation of the FM phase. The latter, in accordance with the Arrhenius equation, must be proportional to $\exp (-\frac{{E}_{{\rm{a}}}}{{kT}})$, where *k* is the Boltzmann constant and ${E}_{{\rm{a}}}$ is the activation energy for the nucleation to occur^43^. In the case of FeRh, ${E}_{{\rm{a}}}$ is a function of *H*. Plotting *A*_L_ and *B*_M_ as 3D graphs (Fig. 4a, b) we see qualitative agreement with the Arrhenius equation – the amplitudes increase with increasing either the temperature or the magnetic field strength. This trend is observed as long as most of the probed FeRh volume is in the AFM state. Approaching the critical magnetic field and temperature, co-existing FM domains will start to dominate the signal and the trend changes completely. All these observations point out that the amplitudes *A*_L_ and *B*_M_ serve as a measure of the volume, which undergoes structural and magnetic changes at the phase transition from the AFM to the FM states.

If one assumes that the transient reflectivity probes structural dynamics of FeRh, the rise time of τ_L_ = 2 ps is expected to be close to the time of expansion of the studied sample area, which can be roughly estimated as ${\tau }_{{\rm{L}}}\approx \frac{{d}_{{\rm{depth}}}}{v}$, where *v* ≈ 4.8 x10^3 ^m/s is the speed of sound as estimated in ref. ^44^ and ${d}_{{\rm{depth}}}$ is the penetration depth of the probe light in the film at the probe wavelength. According to ref. ^22^ the penetration depth for pump and probe pulses in our experiment is ~15 nm. If one takes into account the thickness of the capping Pt layer, we obtain *d*_depth_ ≈ 10 nm, leading to ${\tau }_{{\rm{L}}}\approx \frac{{d}_{{\rm{depth}}}}{v}\approx 2.1$ ps, which is indeed close to the value we found experimentally. It is unclear, however, if the time required for the expansion should also account for the thickness of the Pt capping layer. One can argue that the laser pulse launches an expansion wave at the Pt-vacuum interface and it will take more than 1 ps for this expansion wave to arrive at the Pt-FeRh interface. Only afterwards reflectivity data will contain information about the lattice dynamics of FeRh. We argue, however, that our experiments do not support this hypothesis. For instance, at higher temperatures, when femtosecond laser pulses launch ultrafast demagnetization of the FM-phase, or in higher magnetic fields, when the fastest possible magnetization dynamics in the AFM phase is observed, the signals of transient reflectivity and p-MOKE start their rises practically simultaneously. As the p-MOKE, in contrast to reflectivity, is dominated by the magnetization of Fe-spins, based on these observations we tend to conclude that femtosecond laser pulse launches both reflectivity and p-MOKE dynamics upon reaching FeRh/Pt interface. The fitting and averaging procedures gave very close values for the characteristic rise times for the laser-induced p-MOKE and reflectivity signals: ${\tau }_{{\rm{M}}}=2.2\,\pm 0.4$ ps, *τ*_L_ = 2.7 ± 0.9 ps.

In addition, we observed a magnetic field-independent (damped) oscillatory signal in both p-MOKE and reflectivity channels. A detailed discussion of the origin of the oscillations is beyond the scope of this paper. In any case, even from the experimental raw data, it is directly evident that under strong magnetic fields ${{\tau }_{{\rm{M}}}\approx \tau }_{{\rm{L}}}$. Note that previous studies of the phase transition with the help of X-ray techniques reported similar characteristic times of the lattice expansion. The latter was shown to be defined by the speed of sound and the thickness of the film. Reference ^22^ reported a shift of the Bragg peak already within the first 6 ps for a 47 nm thick film, while in ref. ^45^ similar measurements on a film with a thickness of 100 nm showed a shift of the Bragg peak after 18.5 ps. XAS measurements done in a 30 nm thick FeRh film demonstrated changes on a sub-10 ps time scale^20^. Thus, in the case of the film with a thickness of 40 nm, the lattice expansion should result in a reflectivity change on a sub-10 ps time scale as well. Applying even higher fields is not expected to accelerate the lattice dynamics and, obviously, does not accelerate the spin dynamics. It suggests that the lattice expansion and the magnetization emergence occur simultaneously on a sub-10 ps time scale.

The observed magneto-structural dynamic correlations suggest two possible scenarios. As the essence of the net magnetization induced in the FM state is the angular momentum, conservation of the angular momentum must play in these scenarios the central role. In the first scenario, the effective antiferromagnetic exchange interaction acting between the two iron sublattices turns into a ferromagnetic one much faster than 2 ps. Despite this fact, the observed magnetization dynamics occurs on the timescale of the lattice dynamics. It happens because the magnetization dynamics is practically defined by the rate at which the lattice can exchange angular momentum with the spins and accommodate this angular momentum through the processes of structural dynamics^46,47^. Indeed, recent experiments revealed lattice dynamics upon ultrafast demagnetization of ferromagnetic Ni and Fe. The findings imply that the exchange of angular momentum between the lattice and spins at the sub-ps time scale is quite realistic^46,47^. In the second scenario, the exchange interaction and, consequently, the effective field of the exchange interaction ${{\bf{H}}}_{{\rm{E}}}$ changes on the scale of the lattice expansion. In this case, the response of the spins as well as the exchange of angular momentum are fast enough to follow the changes of the exchange field. Evidence showing which scenario actually takes place can be obtained by performing time-resolved measurements of the exchange fields ${{\bf{H}}}_{{\rm{E}}}$. The latter represents a challenging topic for future studies of ultrafast phase transition in FeRh. It is believed that time-resolved RIXS can, in principle, provide information about dynamics of the exchange interaction^48^. However, we are not aware of any experimental technique which enables such measurements with sufficient time and energy resolutions in order to reveal ultrafast dynamics of the exchange field ${{\bf{H}}}_{{\rm{E}}}$ in FeRh^49^. Nevertheless, regarding the demonstrated ability of the lattice in metallic Ni to accommodate the angular momentum of spins at the sub-ps time scale^47^, we are very much in favour of the second scenario, when the exchange interaction and the effective field of the exchange interaction ${{\bf{H}}}_{{\rm{E}}}$ change on the scale of the lattice expansion.

By analysing laser-induced dynamics in an unprecedentedly broad range of magnetic fields and temperatures we found the conditions corresponding to the fastest possible laser-induced spin dynamics in our FeRh sample. We showed that the emergence of the ferromagnetic phase can only be accelerated to a certain limit, which does not depend on magnetic field or temperature. We argue that the magnetism-or-lattice causality dilemma in the discussion of the mechanism of the magneto-structural phase transition in FeRh can be resolved by the simultaneous evolution of both the actors. Interestingly, the recent ultrafast X-ray diffraction studies of the phase transition in FeRh^50^ also claim that the laser-induced dynamics has an intrinsic time-scale of 8 ps. This value is of the same order of magnitude as the limit revealed in our experiments. Most importantly, our results show that even in magnetic fields as high as 25 T—where the Larmour precession of an electron approaches the terahertz frequency benchmark, the fastest route for AFM-to-FM phase transition in FeRh takes longer than 1 ps. Consequently, our work demonstrates that previously reported laser-induced terahertz emission from FeRh is not due to ultrafast laser-induced ferromagnetism in the antiferromagnet and must be assigned to other mechanisms such as ultrafast expansion or ultrafast demagnetization of domains of the ferromagnetic phase in antiferromagnetic FeRh.

## Methods

### Ultrafast time-resolved pump-probe experiments in high magnetic fields

We employed time-resolved magneto-optical measurement setup at the High Field Magnetic Laboratory in Nijmegen^28^. An amplified Ti:Sapphire laser and an optical parametric amplifier were employed as ultrashort light sources. The studied sample was placed in a cryostat and a Florida-Bitter magnet. The 800 nm (1.55 eV) pump pulse fluence was fixed to 4.1 mJ/cm^2^ and focused on to sample surface with a spot size of ~50 µm in diameter. The pump-induced change in magnetization (Δθ_K_) was observed, via the p-MOKE, using 600 nm (1.9 eV) probe pulses derived from the OPA. Both pump and probe pulses arrived on to the sample surface at normal incidence (see Fig. 1a). Simultaneously, we also recorded the pump-induced reflectivity change (ΔR). The estimated duration of the optical pulses at the sample was around 150 fs. The static magnetic characterization of the sample was done using both p-MOKE and SQUID magnetometry (see Supplementary Materials Figs. S1) in high magnetic fields (up to 25 T). Note that in all the experiments the external magnetic field was applied along the sample normal only. Considering the specific heat of FeRh as 0.35 J/g K^33^, for the pump laser fluence used in our experiments, the sample absorbs about 20% of the pump radiation^51^, we estimate that a single pump laser pulse heats up the upper 10 nm layer by about 230 K.

### Sample and its characterization

The studied sample was a 40 nm thick, epitaxial Fe_50_Rh_50_ film deposited onto an MgO(001) single crystal substrate and then capped with a 5 nm thick Pt layer. The total volume of the FeRh(001) layer was estimated to be 2.9 × 10^−7^ cm^3^. More details regarding the structural characterization of the studied film, including X-ray reflectometry, X-ray diffraction, and manufacturing procedure, are reported in ref. ^42^. The net magnetic moment *M* measured in emu was recalibrated to the magnetic moment per unit cell *μ* in the units of the Bohr magneton ${\mu }_{B}$, using $\mu =\frac{M\left[{emu}\right]{m}_{{mol}}[g/{mole}]}{{\mu }_{B}{N}_{A}m[g]}$, where *N*_A_ is Avogadro’s number, *m*_mol_ is the molar mass and *m* is the mass of the FeRh film.

### Numerical calculation of the H-T phase diagram for FeRh

In order to estimate ${H}_{{\rm{sf}}}$ for FeRh, we write the corresponding thermodynamic potential similar to the one used in ref. ^27^ but upgraded with terms accounting for magnetic anisotropy and the interaction of spins with the external magnetic field:

3 $$\begin{array}{l}{F}_{{eq}}=-\left({J}_{{\rm{Fe}}-{\rm{Fe}}}^{\left(2\right)}\left(T\right)+\frac{{\rho }_{{\rm{J}}}^{2}}{2\epsilon }\right){\left({{\bf{M}}}_{1}\,\cdot\, {{\bf{M}}}_{2}\right)}^{2}-{J}_{{\rm{Fe}}-{\rm{Fe}}}^{\left(1\right)}(T){{\bf{M}}}_{1}\,\cdot\, {{\bf{M}}}_{2}-{{\bf{H}}}_{0}\left({{\bf{M}}}_{1}+{{\bf{M}}}_{2}\right)\\\qquad\quad+\,K(T)V/2\left(\frac{{\left({{\bf{M}}}_{1}\,\cdot\, \hat{{\bf{z}}}\right)}^{2}}{{{\bf{M}}}_{1}^{2}}+\frac{{\left({{\bf{M}}}_{2}\,\cdot\, \hat{{\bf{z}}}\right)}^{2}}{{{\bf{M}}}_{2}^{2}}\right)\end{array}$$

Here ${{\bf{M}}}_{1},\,{{\bf{M}}}_{2}\,(\left|{{\bf{M}}}_{1}\right|=\left|{{\bf{M}}}_{2}\right|={\rm{M}}\approx \,1.5\,{{{\mu }}}_{{\rm{B}}}\,per\, unit\, cell)$ are the magnetizations of the two iron sublattices with opposite spin in the AFM phase. In the first term ${\rho }_{{\rm{J}}}$ is a lattice-dependent exchange constant as proposed in ref. ^7^, $\epsilon$ is the stiffness constant^7,30,52^ and ${J}_{{\rm{Fe}}-{\rm{Fe}}}^{\left(2\right)}\left(T\right)$ is an effective biquadratic iron-iron exchange constant as introduced in refs. ^38,39^. The second term is given by Heisenberg exchange with ${J}_{{\rm{Fe}}-{\rm{Fe}}}^{\left(1\right)}(T)$ being the temperature-dependent isotropic Heisenberg iron-iron exchange constant. The third term defines the interaction of ${{\bf{M}}}_{1}$ and ${{\bf{M}}}_{2}$ with the magnetic field ${{\bf{H}}}_{0}$ applied along the z-axis (see Fig. 2a). The last term describes the magnetic anisotropy, with $K(T)$ is the constant of magnetic anisotropy, which is, in principle, temperature dependent. In this case, the anisotropy is defined such that it favours alignment of ${{\bf{M}}}_{1}$ and ${{\bf{M}}}_{2}$ along the z-axis. $V$ is the unit cell volume.

Introducing the angle $\psi$ as defined in Fig. 2a, we describe the orientations of the magnetizations of the Fe sublattices (${{\bf{M}}}_{{\bf{1}}}$ and ${{\bf{M}}}_{{\bf{2}}}$) with respect to the magnetization **M=(M**_**1**_ + **M**_**2**_**)**/2. $\theta$ is the angle formed by the antiferromagnetic vector **L** **=** **(M**_**1**_ − **M**_**2**_)/2 and the z-axis. In the case of the “easy-axis” of magnetic anisotropy considered here, one gets ${KV}\, < \,0,$ antiferromagnetic coupling favoured by the two-spin exchange interaction ${J}_{{\rm{Fe}}-{\rm{Fe}}}^{\left(1\right)}\left(T\right)\, < \,0$, and ferromagnetic coupling favoured by the second exchange term ${{J}_{2}=J}_{{\rm{Fe}}-{\rm{Fe}}}^{(2)}+\frac{{\rho }_{{\rm{J}}}^{2}}{2\epsilon }\, > \,0$.

Minimization of this total free energy with respect to the angles *ψ* and *θ* gives the conditions for the equilibrium states. The collinear AFM phase corresponds to $(\psi ={\rm{\pi }}/2,\theta =0)$. In the canted AFM phase in an external magnetic field equal to the spin-flop field ${H}_{{\rm{sf}}}$ one can just expect $\psi ={\psi }_{{\rm{sf}}},\theta ={\rm{\pi }}/2$, where $\cos {\psi }_{{\rm{sf}}}\,\ll \,1$. Hence neglecting terms of higher order, such as those $\sim {\cos }^{4}\psi$, at the spin-flop field the free energy in the canted AFM phase can be simplified to:

4 $${{\rm{F}}}_{{\rm{sf}}}\left({\psi }_{{\rm{sf}}}\right)\approx \left(4{J}_{2}{M}^{4}-2{J}_{{\rm{Fe}}-{\rm{Fe}}}^{\left(1\right)}{M}^{2}+{KV}\right){\cos }^{2}{\psi }_{{\rm{sf}}}-2{H}_{{sf}}M\cos {\psi }_{{\rm{sf}}}-{J}_{2}{M}^{4}+{J}_{{\rm{Fe}}-{\rm{Fe}}}^{\left(1\right)}{M}^{2}$$

As the thermodynamic equilibrium implies $\frac{\partial {{\rm{F}}}_{{\rm{sf}}}\left({\psi }_{{\rm{sf}}}\right)}{\partial {\psi }_{{\rm{sf}}}}=0$, it is easy to find the spin-flop angle:

5 $$\cos {\psi }_{{\rm{sf}}}=\frac{{H}_{{\rm{sf}}}}{2{H}_{{\rm{E}}}-{H}_{{\rm{A}}}},$$

where ${J}_{{\rm{eff}}}={J}_{{\rm{Fe}}-{\rm{F}}e}^{(1)}-2\left({J}_{{\rm{Fe}}-{\rm{Fe}}}^{(2)}+\frac{{\rho }_{{\rm{J}}}^{2}}{2\epsilon }\right){M}^{2}$, ${H}_{{\rm{E}}}\equiv -{J}_{{\rm{eff}}}M$, and the anisotropy field ${H}_{{\rm{A}}}=-\frac{{KV}}{M}$.

The free energy in the collinear AFM state is equal to

6 $${F}_{{col}}=-{J}_{2}{M}^{4}+{J}_{{\rm{Fe}}-{\rm{Fe}}}^{(1)}{M}^{2}+{KV}$$

The spin-flop field ${H}_{{sf}}$ is defined as the field for which ${{\rm{F}}}_{{\rm{sf}}}\left({\psi }_{{\rm{sf}}}\right)={{\rm{F}}}_{{\rm{col}}}$. Thus, comparing the free energies of the canted and the collinear AFM phases, given by Eq. (4) and Eq. (6), one finds:

7 $${H}_{{\rm{sf}}}=\sqrt{({H}_{{\rm{A}}}/2)(2{H}_{{\rm{E}}}-{H}_{{\rm{A}}})}\,\approx \sqrt{{H}_{{\rm{A}}}{H}_{{\rm{E}}}}$$

Using Eqs. (5) and (7) we can estimate ${H}_{{\rm{sf}}}$ and ${\psi }_{{\rm{sf}}}$ assuming ${J}_{{\rm{eff}}}$ of the order reported in ref. ^11^. Performing numerical minimization of the thermodynamic potential given by Eq. (3), we can also analyze the whole *H-T* phase diagram. We assumed that$\,{J}_{{\rm{eff}}}(T)$ is linear with *T* and changes sign at the transition temperature^41^, and the uniaxial anisotropy is out-of-plane. We note, however, that there is no experimental data on the type and strength of the magnetic anisotropy in the AFM phase of FeRh. However, thin magnetic films with zero magnetization, such as ferrimagnets at the compensation temperature, favour out-of-plane magnetic anisotropy due to the absence of demagnetizing fields and a dominant surface anisotropy contribution, resulting from the breaking of the inversion symmetry at the interfaces of the film. Such out-of-plane anisotropy in the AFM phase of FeRh is also predicted by computational studies in refs. ^53,54^. Hence we assumed that the value for the constant of magnetic anisotropy *K* is of the order of those proposed in refs. ^54–56^. To further simplify the model, we assume that the magnetic anisotropy is temperature-independent. The actual parameters used in the thermodynamic potential are given by: ${J}_{{\rm{Fe}}-{\rm{F}}e}^{(1)}{M}^{2}=-0.46\,\times {10}^{-14}\left({erg}\right)$, ${J}_{2}{M}^{4}=0.23\ \times{10}^{-14}\left({erg}\right)$, $K=5\,\times {10}^{6}({erg}/{cc})$ and the resulting *H-T* phase diagram is shown in Fig. 2a. We see that the model reproduces all three expected phases: collinear AFM (light blue area), canted AFM (dark blue area), and FM (brown area). The calculated critical fields of the phase transition to the FM state (transition from blue to brown areas) are remarkably close to those obtained in our experiment (see Fig. 3a), although in our model for most applied fields this transition to the FM phase starts from the canted AFM phase, rather than from the collinear phase. Only in the lower field region the model predicts a direct transition from the collinear AFM phase to the FM phase. We note that the spin-flop transition (from the collinear AFM phase to the canted AFM phase) has not yet been reported for FeRh, but we envisage that its experimental observation must be seriously hampered by the relatively small angle of the spin canting just after the spin-flop ${\psi }_{{\rm{sf}}}={{5}^{\circ}} -{{10}^{\circ}}$, estimated using our model.

One can argue that there is no real proof of the fact that in our experiment FeRh has a transition between collinear and canted AFM phases. However, at the same time, there is also no experimental proof that the magnetic anisotropy of antiferromagnetic FeRh is in-plane. In this case, the fields applied in our experiment would tilt the spins even easier, but this canting has also never been reported experimentally.

## Supplementary information

Supplementary information

## Data Availability

No datasets were generated or analysed during the current study.
