# Supplementary material for: Ultrafast emergence of ferromagnetism in antiferromagnetic FeRh in high magnetic fields
Source: Npj Spintron. 2025 Feb 3;3(1):5. doi: 10.1038/s44306-024-00069-6 (PMC11790480; doi:10.1038/s44306-024-00069-6)
Supplement: Supplementary file 1 — Supplementary information [file 44306_2024_69_MOESM1_ESM.docx]

**Supplementary information**

**Ultrafast Emergence of Ferromagnetism in Antiferromagnetic FeRh in High Magnetic Fields**

I. A. Dolgikh^1,2,^*, T. G. H. Blank^1^, A. G. Buzdakov^3^, G. Li^1^, K. H. Prabhakara^1^, S. K. K. Patel^4^, R. Medapalli^4^, E. E. Fullerton^4^, O. V. Koplak^5^, J. H. Mentink^1^, K. A. Zvezdin^6^, A. K. Zvezdin^7^, P. C. M. Christianen^2^, and A. V. Kimel^1^

*^1^Radboud University, Institute for Molecules and Materials, 6525 AJ Nijmegen, The Netherlands*

*^2^High Field Magnet Laboratory (HFML - EMFL), Radboud University, Toernooiveld 7, 6525 ED Nijmegen, The Netherlands*

*^3^Interactive Fully Electrical Vehicles Srl, 10040 La Loggia TO, Italy*

*^4^Center for Memory and Recording Research, University of California San Diego, La Jolla, California 92093-0401, USA*

*^5^University of Milan-Bicocca, Milan, Italy*

^6^Istituto P.M. Srl, 10138 Turin, Italy

*^7^The Lebedev Physical Institute of the Russian Academy of Sciences, 119991 Moscow, Russia*

*email: [i.dolgikh@tue.nl](mailto:i.dolgikh@tue.nl)


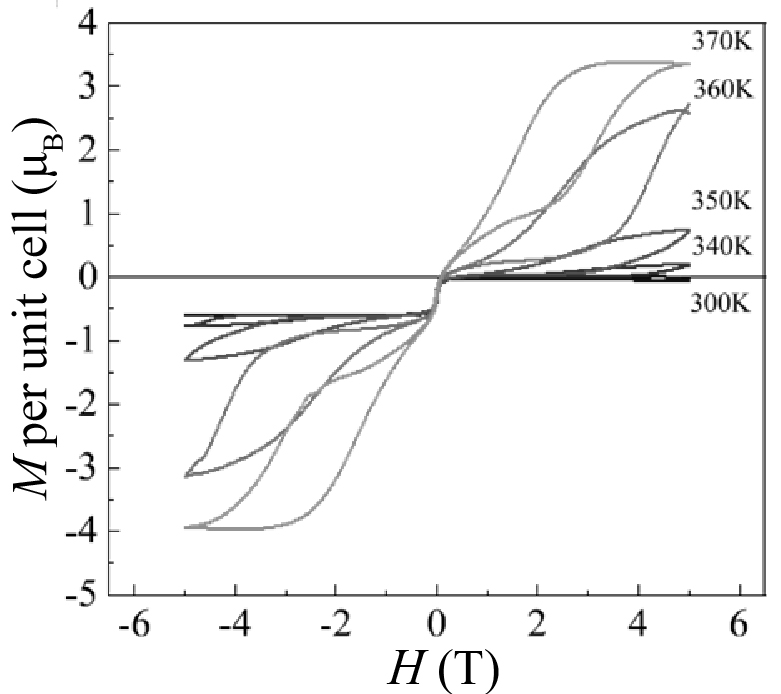


Supplementary Figure 1: Net magnetization curves measured using SQUID magnetometer are shown for different temperatures of the sample ranging between 300 and 370 K.


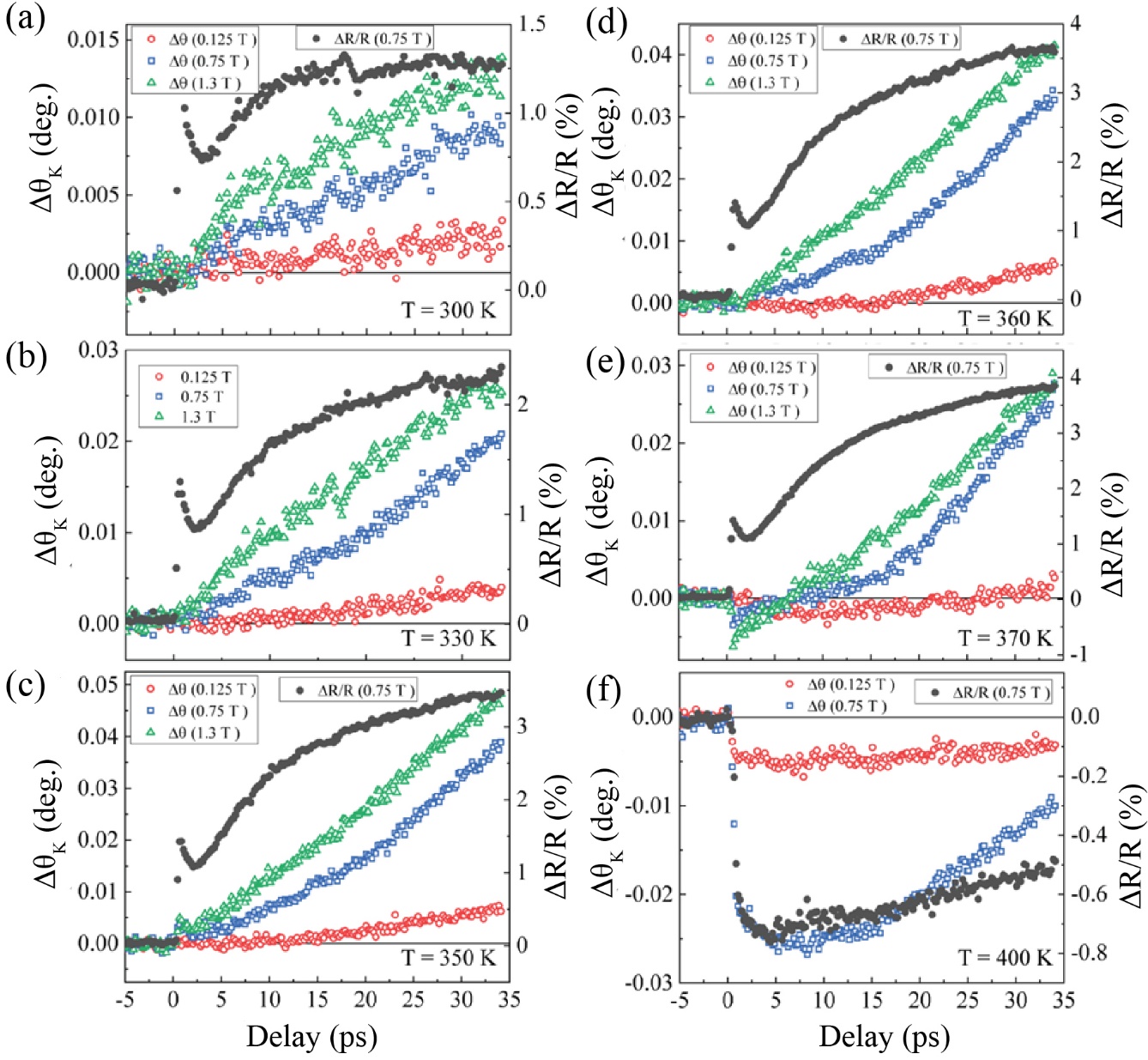


Supplementary Figure 2. Laser-induced ultrafast dynamics measured across the heating cycle of magneto-structural phase transition (300 K to 400 K) in FeRh. Both the laser-induced ΔR (solid black circles) and Δθ_K_ dynamics for three different H values: 0.125 T (circles), 0.75 T (squares), and 1.3 T (triangles) measured at different initial temperatures of the sample are shown.


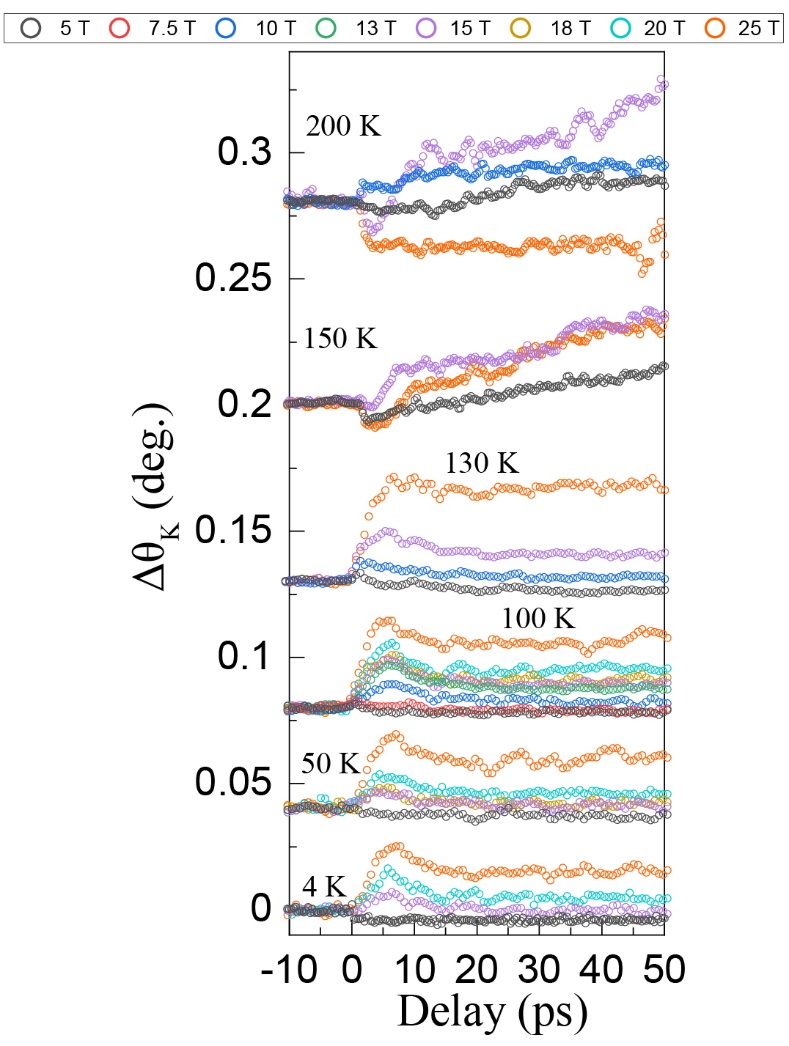


Supplementary Figure 3. Laser-induced ultrafast dynamics, are shown up to 50 ps after the laser excitation, for different combinations of (*H* and *T*) values. The curves are plotted temperature wise with an offset along the y-axis.

**
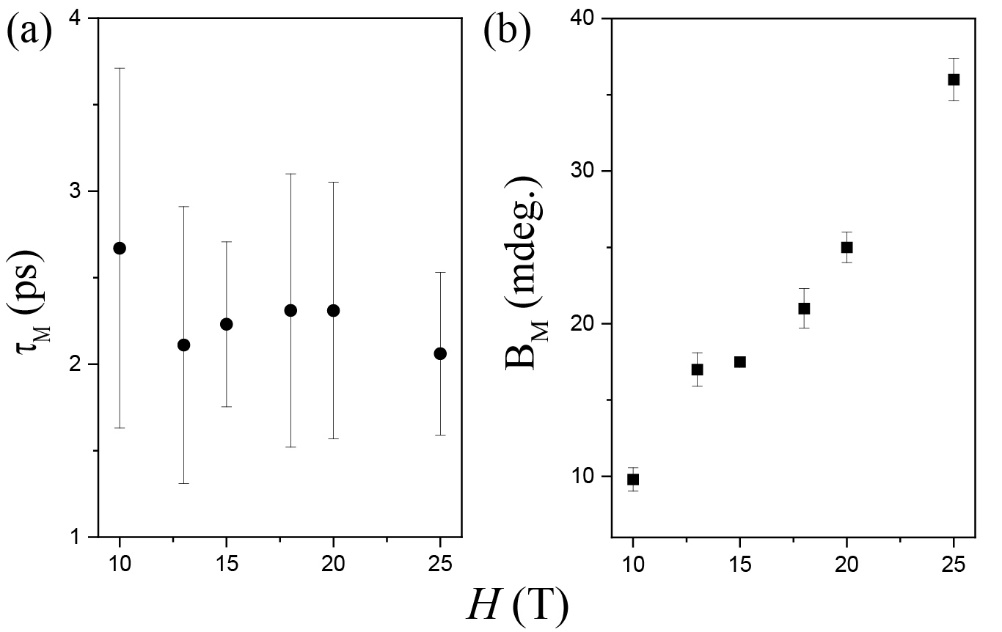
**

Supplementary Figure 4. Fitting parameters of the laser-induced p-MOKE signals measured for the case *T* = 100 K using Eq.1 given in the main text. Magnetic field dependence of the characteristic rise time of the FM nucleation (a), and the amplitudes of the exponential rise (b) are shown.


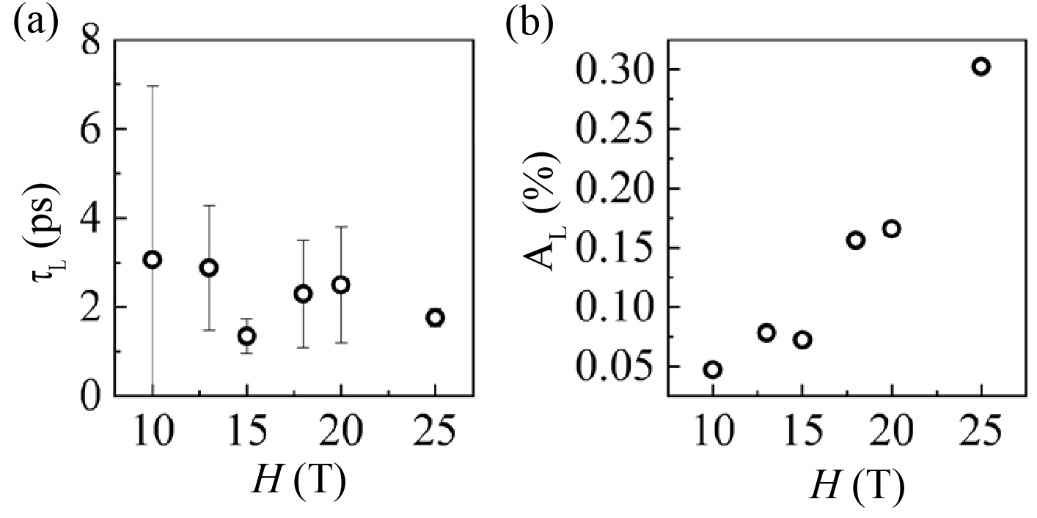


Supplementary Figure 5. Fitting parameters of the laser-induced reflectivity signals measured for the case *T* = 100 K using Eq.2 given in the main text. Magnetic field dependence of the characteristic rise time of the exponential time (a), and the corresponding amplitudes (b) are shown.

**Simulation of ultrafast magnetization dynamics using two spin model**

While modelling ultrafast magnetization dynamics in FeRh remains to be a challenge, it is possible to show that a sudden change of the exchange interaction in collinear and canted antiferromagnetic state triggers substantially different dynamics even in a simple case of two antiferromagnetically coupled macrospins. To this end, we consider a simplified free energy F that accounts for the exchange interactions between two macrospins **M**_1_ and **M**_2_. The latter mimic the two antiferromagnetically coupled Fe-sublattices in FeRh. The free energy also accounts for the interaction of the applied magnetic field $\mathbf{H}_{0}$ with the macrospins as well as assumes that the field is applied along the easy axis of magnetic anisotropy (*z*-axis) with uniaxial anisotropy constant *K*:

| $F=f\left( \mathbf{M}_{1}^{2} \right)+f\left( \mathbf{M}_{2}^{2} \right)-J\mathbf{M}_{1}\cdot\mathbf{M}_{2}-\mathbf{H}_{0}\cdot\left( \mathbf{M}_{1}+\mathbf{M}_{2} \right)+K\left( \frac{\left( \mathbf{M}_{1}\cdot\hat{z} \right)^{2}}{\mathbf{M}_{1}^{2}}+\frac{\left( \mathbf{M}_{2}\cdot\hat{z} \right)^{2}}{\mathbf{M}_{2}^{2}} \right)$ | (6) |
| --- | --- |

here $f\left( \mathbf{M}_{1}^{2} \right)$ and $f\left( \mathbf{M}_{2}^{2} \right)$ are the non-equilibrium exchange energy of sublattices, as defined Ref. [1], and *J* is the exchange constant that favours antiferromagnetic coupling between the macrospins in the ground state.

The dynamics of the macrospins is described by the set of equations describing both longitudinal and transverse spin dynamics [2,3]:

| $\frac{\hbar}{\gamma}\frac{d\mathbf{M}_{1}}{dt}=\mathbf{M}_{1}\times\mathbf{H}_{1}+\lambda_{r}\mathbf{H}_{1}+\lambda_{e}\left( \mathbf{H}_{1}-\mathbf{H}_{2} \right),$ | (7) |
| --- | --- |
| $\frac{\hbar}{\gamma}\frac{d\mathbf{M}_{2}}{dt}=\mathbf{M}_{2}\times\mathbf{H}_{2}+\lambda_{r}\mathbf{H}_{2}+\lambda_{e}\left( \mathbf{H}_{2}-\mathbf{H}_{1} \right),$ | (8) |

| $\mathbf{H}_{1,2}=-A\left( \mathbf{M}_{1,2}^{2}-\mathbf{M}_{0}^{2} \right)\mathbf{M}_{1,2}+J\mathbf{M}_{2,1}+\mathbf{H}_{0}-2K\frac{\mathbf{M}_{1,2}\cdot\hat{z}}{\mathbf{M}_{1,2}^{2}}.$ | (9) |
| --- | --- |

where the effective fields $\mathbf{H}_{i}\equiv-\frac{\delta F}{\delta\mathbf{M}_{i}}$ are derived from the free energy *F* given by Eq. 7 [1]:

Here *A* is a non-equilibrium exchange constant [1] and M_0_ is the saturation magnetization of the iron sublattices. The relativistic constant *λ*_r_ describes angular momentum transfer to the environment, *λ*_ε_ allows for exchange-mediated transfer between the sublattices, *γ* is the gyromagnetic ratio of iron.

In the model, the action of the laser pulse is assumed to change the sign of the exchange constant *J* → –*J* within 30 fs. Parameters used for modelling are *J* = –24.8 meV, *K* = 9.75×10­^-5^ J, A = 4 J, M_0_ = 0.1, *λ*_ε_ = 0.05, *λ*_r_ = 0.2 *λ*_ε_ [4-6] The magnetization of the macrospins, as well as the relaxation parameters, are dimensionless and, the former is set to unity. The model gives for the spin-flop field ${\mu_{0}H}_{\mathrm{sf}}=$6 T. The results of the simulations of the magnetization dynamics are depicted in Fig.S12 for the cases of low magnetic field ${\mu_{0}H}_{0}=$0.6 T and the field above the spin-flop field $\mu_{0}H_{0}=$9 T, where the macrospins are initially in a canted antiferromagnetic state. It is clearly shown that a high magnetic field above the spin-flop field accelerates the dynamics in qualitative agreement with our experimental findings. Furthermore, it is possible that FeRh exhibits multi domain and single domain structures under lower and strong applied magnetic fields, respectively. However, we acknowledge that our current simple two-spin model does not account for the domain structure effects, as it is designed to capture the essential spin dynamics rather than the complexities of domain behavior. We believe this simplified approach is still valid for understanding the fastest spin dynamics observed during the canted AFM – FM phase transition.


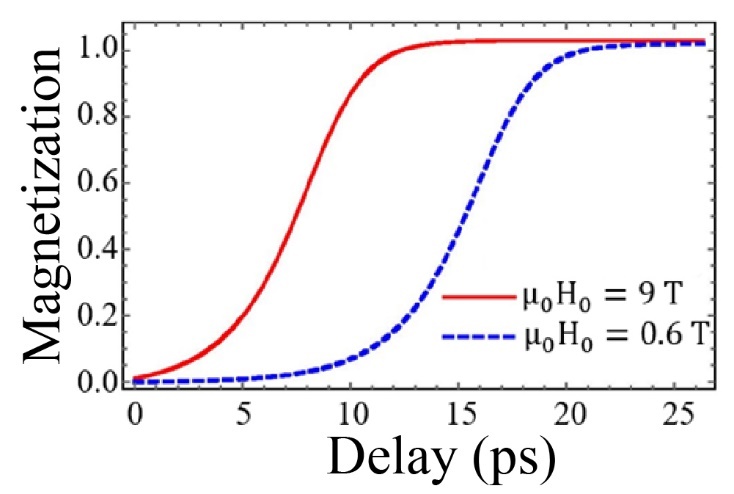


Supplementary Figure 6. Simulated magnetization dynamics of a two-sublattice antiferromagnet with ultrafast exchange-inversion occurring at zero-time delay. Using the set of parameters as explained in the text, the system has a spin-flop transition at $\mu_{0}H_{\mathrm{sf}}=6 T$. When starting far below this field (0.6 T), the modelled dynamics shows a clear latency period in the growth of the magnetization along the applied field. This is qualitatively different from the situation in high fields ($\mu_{0}H_{\mathrm{sf}}=9 T$), where the system initially resides in the canted antiferromagnetic state.

**References**

[1] J. H. Mentink, Magnetism on the Timescale of the Exchange Interaction: Explanations and Predictions Interaction, Radboud Universiteit Nijmegen, 2012.

[2] J. H. Mentink, J. Hellsvik, D. V. Afanasiev, B. A. Ivanov, A. Kirilyuk, A. V. Kimel, O. Eriksson, M. I. Katsnelson, and Th. Rasing, *Ultrafast Spin Dynamics in Multisublattice Magnets*, Phys. Rev. Lett. **108**, 057202 (2012).

[3] V. G. Bar’yakhtar, V. I. Butrim, and B. A. Ivanov, *Exchange Relaxation as a Mechanism of the Ultrafast Reorientation of Spins in a Two-Sublattice Ferrimagnet*, JETP Lett. **98**, 289 (2013).

[4] J. Barker and R. W. Chantrell, *Higher-Order Exchange Interactions Leading to Metamagnetism in FeRh*, Phys. Rev. B **92**, 094402 (2015).

[5] G. Ju, J. Hohlfeld, B. Bergman, R. J. M. van de Veerdonk, O. N. Mryasov, J.-Y. Kim, X. Wu, D. Weller, and B. Koopmans, *Ultrafast Generation of Ferromagnetic Order via a Laser-Induced Phase Transformation in FeRh Thin Films*, Phys. Rev. Lett. **93**, 197403 (2004).

[6] J. Cao, N. T. Nam, S. Inoue, H. Y. Y. Ko, N. N. Phuoc, and T. Suzuki, *Magnetization Behaviors for FeRh Single Crystal Thin Films*, J. Appl. Phys. **103**, 07F501 (2008).
